# Shared and Unique Features Distinguishing Follicular T Helper and Regulatory Cells of Peripheral Lymph Node and Peyer’s Patches

**DOI:** 10.3389/fimmu.2018.00714

**Published:** 2018-04-09

**Authors:** Hristo Georgiev, Inga Ravens, Georgia Papadogianni, Stephan Halle, Bernard Malissen, Gabriela G. Loots, Reinhold Förster, Günter Bernhardt

**Affiliations:** ^1^Institute of Immunology, Hannover Medical School, Hannover, Germany; ^2^Centre d’Immunologie de Marseille-Luminy, Aix Marseille Université, INSERM, CNRS, Marseille, France; ^3^Biology and Biotechnology Division, Lawrence Livermore National Laboratory, Livermore, CA, United States

**Keywords:** follicular helper T cells, follicular regulatory T cells, lymph node, Peyer’s patch, transcriptome, germinal center

## Abstract

Follicular helper (TFH) and regulatory (TFR) cells are critical players in managing germinal center (GC) reactions that accomplish effective humoral immune responses. Transcriptome analyses were done comparing gene regulation of TFH and TFR cells isolated from Peyer’s Patches (PP) and immunized peripheral lymph nodes (pLNs) revealing many regulatory patterns common to all follicular cells. However, in contrast to TFH cells, the upregulation or downregulation of many genes was attenuated substantially in pLN TFR cells when compared to those of PP. Additionally, PP but not pLN TFR cells were largely unresponsive to IL2 and expressed *Il4* as well as *Il21*. Together with fundamental differences in gene expression that were found between cells of both compartments this emphasizes specific adaptations of follicular T cell functions to their micro-milieu. Moreover, although GL7 expression distinguishes matured follicular T cells, GL7^+^ as well as GL7^−^ cells are present in the GC.

## Introduction

In the course of an adaptive immune response, some of the antigen primed CD4 T cells differentiate into follicular helper T (TFH) cells (1). This occurs in a stepwise process (2). Already at the initial contact of a cognate CD4 T cell with an antigen presenting dendritic cell (DC) a first commitment toward TFH differentiation is made as reflected by an early upregulation of key genes of this pathway, such as Bcl6 (3, 4), Ascl2 (5), ICOS (6, 7), PD1 (8), or BATF/c-Maf (9). This is accompanied by the downregulation of CCR7 expression and a concomitant upregulation of CXCR5 guiding the Bcl6^int^PD1^int^ preTFH cells to the T/B border area (2), where they can contact antigen activated B cells. Proper signals received by the cognate B cells then trigger further upregulation of markers, such as BCL6, PD1, ICOS, and CXCR5 completing the TFH cell differentiation program that ultimately leads to the migration of the TFH cells into the B cell follicle and the founding of a germinal center (GC). In contrast, inadequately stimulated preTFH cells could assist B cells fostering an early antibody response whereas cells that failed to come into contact with antigen may develop into TH2 cells (10). Fully matured TFH cells are defined as Bcl6^hi^PD1^hi^ICOS^hi^CXCR5^hi^CCR7^lo^ cells but a wealth of other markers may be included that where shown to be down or upregulated in TFH cells such as KLF2 (11), IL4 (10), or IL21 (12). The final differentiation into GC TFH cells is flexible: the cells may revert to a more immature stage becoming memory cells (13) that can circulate in blood and may give rise to effector helper T and TFH cells in the course of a recall response (14, 15). Moreover, GC TFH cells are not restricted to their GC but can travel to neighboring GC inside the secondary lymphoid organ (16).

The GC reaction is a highly dynamic process and must be tightly controlled in order to prevent the exuberant production of antibodies and autoimmunity. In part, this is accomplished by follicular regulatory T (TFR) cells (17, 18). TFR cells develop from natural regulatory T (Treg) cells and possess a TCR repertoire different from that of the TFH cells originating from the same antigenic challenge (19). Yet phenotypically, TFR cells closely resemble TFH cells. To some degree this is dictated by the need to be present at the same location, the GC, as the cells to be controlled, the GC TFH and B cells. Thus, TFR cells were also described as Bcl6^+^PD1^+^CXCR5^hi^CCR7^lo^ cells but in addition express Foxp3, the master transcription factor managing their inhibitory potential. Like TFH cells, memory TFR cells circulate in blood (20).

Peyer’s Patches (PP) differ from peripheral lymph nodes (pLNs) in several aspects (21): PP do not possess afferent lymphatics but receive antigen directly from the lumen of the gut that is rich in commensal bacteria. Gut contents are transported *via* M cells into the PP and taken up by DC residing in the subepithelial dome that process and present antigen to T cells. The continuous exposure of PP cells to bacterial constituents keeps the GC inside PP under constant operation. On the other hand, the intestinal immune compartment is tolerogenic by nature thus preventing unwanted immune reactivity against otherwise harmless antigens (22). Moreover, the main antibody produced by PP derived plasma cells is IgA whereas switch to an IgG class is usually performed by B cells in pLN where GC emerge only on demand. These differences render it likely that TFH and TFR cells of PP might differ from their counterparts in pLN in handling a GC reaction. This is evidenced by the finding that in PP but not pLN Treg cells can develop *via* a TFR stage into TFH cells (23, 24).

Here, we explored the characteristics of pLN and PP derived TFH as well as TFR cells based on analysis and comparison of their transcriptomes. Expectedly, TFR cells resemble TFH cells but in pLN the follicular phenotype as compared to PP TFR cells remains incomplete regarding many genes. The latter not only gradually differed from pLN TFR cell but also fundamentally in several aspects. PP TFR cells produce IL4 and to some extent also IL21. Moreover, in contrast to pLN TFR cells, PP TFR cell cannot propagate in response to IL2 because they lack expression of CD25. We also show that in PP GC follicular T cells are promiscuous with regard to expression of the GL7 epitope disqualifying it as an exclusive marker for GC resident cells.

## Materials and Methods

### Mice

C57BL/6NCrl (B6) mice were originally purchased from Charles River (Strain number: 027) and B6.Cg-Ptprc^a^ Tg(UBC-PA-GFP)1Mnz Ptprc^a^-Pepc^b^/Boy (PA-GFP) mice from Jackson Laboratories (Strain number: 022486). B6.Cg-Foxp3^<tm1Mal>^ (Foxp3) mice were provided by B. Malissen (Aix-Marseille Université, Marseille, France) and B6.Sostdc1^tm1(KOMP)Vlcg^ (Sostdc1^−/−^) mice by G. Loots (Lawrence Livermore National Laboratory, Livermore, USA). All mice were bred at the central animal facility at Hannover Medical School (MHH) under specific pathogen-free conditions. Females and males, 7- to 12-week-old mice were used in all experiments.

### Immunizations

To induce development of GCs and follicular CD4 T cells, mice were immunized subcutaneously (s.c.) into both flanks with 200 µg keyhole limpet hemocyanin (KLH) mixed with Alhydrogel adjuvant 2% (Alum) at 1:1 v:v ratio in 100 µl volume. Post immunization mice were sacrificed at the indicated time points and draining lymph nodes (iLNs) were harvest for further analysis.

### Flow Cytometry and Cell Sorting

Organs were harvested on ice in FACS buffer (PBS/3% FCS) and single-cell suspensions were prepared by meshing through 40-µm cell strainers. Prior staining samples are blocked with 3% rat serum in FACS buffer. All surface stainings were done on ice for 30 min, except for CCR7 that was performed at 37°C for 30 min. Intracellular cytokine stainings were done using the ICS staining buffer set (eBioscience cat# 88-8824-00) and intracellular detection of FOXP3 was performed by using a FOXP3 staining buffer set (eBioscience cat# 00-5523-00), following the protocol provided by the manufacturer. The complete list of all antibodies used in this study is: anti-mouse CD19 (clone 6D5), anti-mouse CD4 (clone RM4-5), anti-mouse IFN-γ (clone XMG1.2), anti-mouse IL-17A (clone TC11-18H10.1), anti-mouse CD8α (clone 53-6.7), anti-human/mouse CD49f (clone GoH3), anti-mouse CD196 (CCR6) (clone 29-2L17), anti-mouse Syndecan-1 (CD138) (clone 281-2), anti-mouse CD3 (clone 17A2), IgG1, κ Isotype Ctrl Antibody (clone RTK2071), IgG2a, κ Isotype Ctrl Antibody (clone RTK2758), IgG2b, κ Isotype Ctrl Antibody (clone RTK4530), IgG Isotype Ctrl Antibody (clone HTK888) all from BioLegend; anti-human/mouse CD45R (B220) (clone RA3-6B2), anti-mouse CD95 (APO-1/Fas) (clone 15A7), anti-mouse IL10 (clone JES5-16E3), anti-mouse CD279 (PD-1) (clone J43), anti-mouse CD197 (CCR7) (clone 4B12), anti-mouse CD278 (ICOS) (clone 7E.17G9), anti-mouse CD185 (CXCR5) (clone SPRCL5), anti-mouse CD25 (clone PC61.5), anti-human/mouse GL7 (clone GL7), anti-mouse/rat FOXP3 (clone FJK-16s) all from eBioscience; anti-mouse IL-4 (clone 11B11) and anti-mouse T- and B-Cell Activation Antigen (GL7) (clone GL7) from BD Bioscience; anti-rat/mouse CD29 (Itgb1) (clone HMß11) from Miltenyi Biotec. Anti-mouse IgD (clone HB250) was in-house produced. Data were acquired on LSR II (Becton and Dickinson) and analyzed with FlowJo version 10.1r5 (Tree Star). For cell sorting, single-cell suspensions from pooled pLNs or PP were prepared and stained as described above. Cells were sorted into RPMI 1640/10% FCS medium utilizing FACSAria Fusion (Becton Dickinson) or MoFlo XDP (Beckman-Coulter) cell sorters. Cell fractions are pre-gated on singlets and collected as follows: Naïve CD4 T cells (DAPI^−^B220^−^CD4^+^GFP^−^PD1^−^GL7^−^); TFH GL7^−^ (DAPI^−^B220^−^CD4^+^GFP^−^PD1^hi^GL7^−^); TFH GL7^+^ (DAPI^−^B220^−^CD4^+^GFP^−^PD1^hi^GL7^+^); Tregs (DAPI^−^B220^−^CD4^+^GFP^+^PD1^−^GL7^−^); and TFR cells (DAPI^−^B220^−^CD4^+^GFP^+^PD1^hi^GL7^+/−^).

### *In Vitro* Cytokine Production

Organs were harvested in RPMI 1640/10% FCS medium supplemented with 10 µM KN-62. Total cell preparations were plated at density of 5 × 10^6^/ml and incubated at 37°C for 4 h in RPMI 1640/10% FCS medium supplemented with 10 µM KN-62, Ionomycin 1.5 µg/ml (Invitrogen cat# I-24222), PMA 50 ng/ml (Calbiochem cat# 524400) and Brefeldin A 10 µg/ml (Sigma-Aldrich cat# B6542). During this step control cells are kept on ice in FACS buffer. Following incubation samples were processed for intracellular cytokine stainings by flow cytometry as described above.

### *In Vitro* Cell Proliferation Assay

Peyer’s Patches and pLNs (d12 p.i. with KLH + Alum) were harvested in RPMI 1640/10% FCS medium supplemented with 10-μM KN-62. Single-cell suspensions were prepared and TFH, TFR, and Treg cells were sorted according to the gating strategy depicted in Figures S1A,B in Supplementary Material. Sorted cells were labeled with Cell Proliferation Dye eFluor^®^ 670 (eBioscience cat# 65-0840-90) following the guidance provided from the manufacturer. Following labeling cells were plated in RPMI 1640/10% FCS medium supplemented with recombinant IL2 100U/l at a density of 5 × 10^5^/ml into wells that were coated with 1 µg/ml anti-mouse CD3 antibody (clone 17A2). 4 days post plating, cells were analyzed with the use of flow cytometry for frequencies of proliferated cells.

### Enzyme-Linked Immunosorbent Assay (ELISA)

For analysis of IgA titers in fecal samples, specimens were collected at two different time points from each animal separately. ELISA plates were coated overnight at 4°C with 500 ng goat anti-mouse IgA (BIO-RAD cat# StAR137) in PBSd per well. Weight of stool samples was determined and PBSd was added to obtain 10% w/v suspensions. The suspensions were centrifuged and supernatant dilutions (as indicated in Figure 7E) were added to the plates and incubated for 1 h at 37°C. For detection of IgA levels, the following secondary antibodies were used: goat anti-mouse kappa light chain antibody HRP conjugated (Merck Millipore cat# AP200P) and goat anti-mouse lambda light chain antibody HRP conjugated (Bethyl cat# A90-121P).

### Isolation of RNA and Microarray Assay

Naïve CD4 T cells, Tregs, TFH, and TFR cells were FACS sorted from pooled PP or pLNs from 8 to 15 mice at day 12 or day 25 post s.c. immunization with KLH + Alum. Following sorting RNA was isolated using the RNeasy Plus Micro Kit (Qiagen).

The Microarray study has been performed by use of refined versions of the Whole Mouse Genome Oligo Microarray 4x44K v2 (Design ID 026655, Agilent Technologies), called “026655AsQuadruplicatesOn4x180K” (Design ID 048306) or “048306On1M_V3” (Design ID 084107). Both Microarray designs are developed by the Research Core Unit Genomics of Hannover Medical School. Microarrays of these design types cover roughly 32,000 murine transcripts. Microarray designs were defined at Agilent’s eArray portal using a 4x180K, or a 1 M design format for mRNA expression as template. All non-control probes of design ID 026655 have been selected to be printed four times onto one area covering 180K probes (yielding in on-chip quadruplicate Features). Control probes required for proper Feature Extraction software operation were determined and placed automatically by eArray using recommended default settings.

25–40 ng of total RNA were used to prepare aminoallyl-UTP-modified (aaUTP) cRNA (Amino Allyl MessageAmp™ II Kit; #AM1753; Life Technologies) as directed by the company (applying one-round of amplification), except that reaction volumes were halved. The labeling of aaUTP-cRNA was performed by use of Alexa Fluor 555 Reactive Dye (#A32756; LifeTechnologies). Prior to the reverse transcription reaction, 1 µl of a 1:100,000 dilution of Agilent’s “One-Color spike-in Kit stock solution” (#5188-5282, Agilent Technologies) was added to a subset of total RNA sample.

cRNA fragmentation, hybridization and washing steps were carried-out as recommended in the “One-Color Microarray-Based Gene Expression Analysis Protocol V5.7,” except that 250–300 ng of each fluorescently labeled cRNA population were used for hybridization.

Slides were scanned on the Agilent Micro Array Scanner G2565CA (pixel resolution 3 µm, bit depth 20). Data extraction was performed with the “Feature Extraction Software V10.7.3.1” using the extraction protocol file “GE1_107_Sep09.xml.”

Measurements of on-chip replicates (quadruplicates) were averaged using the geometric mean of processed intensity values of the green channel, “gProcessedSignal” (gPS) to retrieve one resulting value per probe and sample. Single Features were excluded from averaging, if they (i) were manually flagged, (ii) were identified as Outliers by the Feature Extraction Software, (iii) lay outside the interval of “1.42 × interquartile range” regarding the gPS distribution of the respective on-chip replicate population, or (iv) showed a coefficient of variation of pixel intensities per Feature that exceeded 0.5.

Averaged gPS values were normalized by quantile normalization first, followed by global linear scaling. For this latter approach, all gPS values of one sample were multiplied by an array-specific scaling factor. This factor was calculated by dividing a “reference 75th Percentile value” (set as 1,500 for the whole series) by the 75th Percentile value of the particular Microarray to be normalized (“Array I” in the formula shown below). Accordingly, normalized gPS values for all samples (microarray data sets) were calculated by the following formula:

$$\begin{array}{l} \text{normalized gPSArray }i=\text{gPSArray }i\;\\ \times \;\left(\text{1,5}00/\text{75th PercentileArray i}\right). \end{array}$$

A lower intensity threshold (surrogate value) was defined based on intensity distribution of negative control features. This value was fixed to 20. All of those normalized gPS values that fell below this intensity border were substituted by the respective surrogate value of 20.

### Data Availability

RNA data have been deposited in the National Center for Biotechnology Information GEO database (https://www.ncbi.nlm.nih.gov/geo) under the GEO accession number GSE106593.

### Two-Photon Photoactivation and Epifluorescence Microscopy

For *in situ* photoactivation, B6.Cg-Ptprc^a^ Tg(UBC-PA-GFP)1Mnz Ptprc^a^-Pepc^b^/Boy (PA-GFP) mice were sacrificed, PP were explanted and immobilized serosal side facing upwards on a plastic Petri dish using tissue adhesive glue (Surgibond). In total, 7 PP per mouse were explanted: three were kept on ice in FACS buffer as controls four were used for photoactivation of a small region in GCs or in the interfollicular region.

A 2-photon laser tuned to 825 nm (with low energy intensity) was used to localize the GC. Beneath the serosal collagen, a layer of autofluorescent macrophages outlines the outer part of the GC as illustrated in Figures 6B,C. Starting directly under the autofluorescent macrophage layer, cuboids with the dimension of 100–200 µm and 40 µm in depth were treated with the 2-photon laser at 825 nm (with high energy intensity). In every PP, three B cell follicles were photoactivated. The size of each cuboid was matched to the size of the follicle, GC orientation and proximity to vessels (Figures 6B,C). For the two-photon treatment of the interfollicular region, a cube with the dimension of 100 µm × 100 µm × 40 µm was converted in the area between two B cell follicles (Figures 6B,C). The treated samples were imaged using a Leica epifluorescence microscope to validate positioning of the treated regions. Finally, single-cell suspensions were prepared from each PP and flow cytometry analysis was performed (Figure 6D).

### Bioinformatics and Statistical Analysis

**Figure 1:**
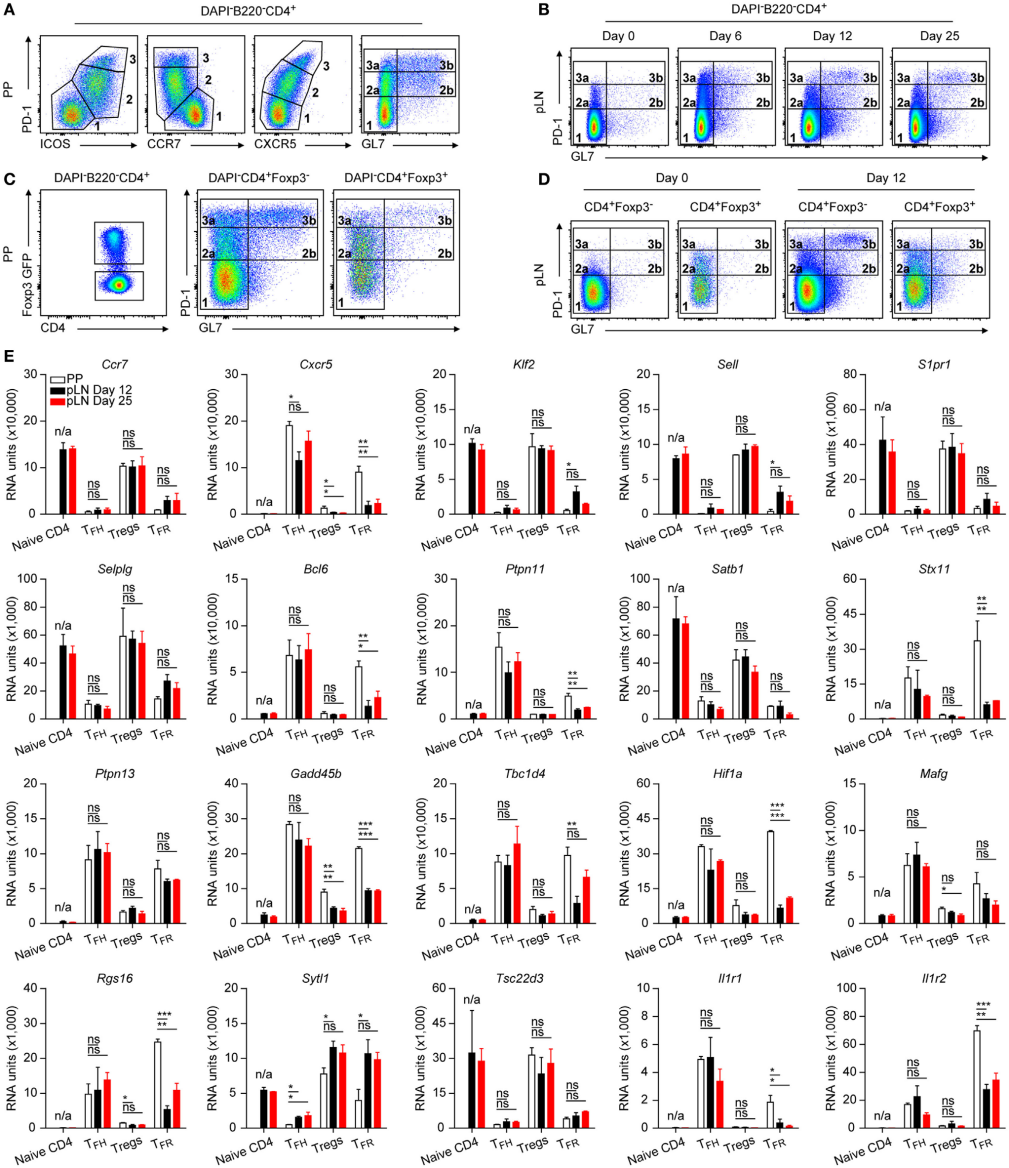
Characterization of follicular CD4 T cells. **(A–D)** Flow cytometry profiles of different T_FH_ cell markers as indicated expressed on CD4 T cells from Peyer’s Patches (PP) and peripheral lymph node (pLN) 6, 12, or 25 days p.i. with KLH + Alum. Data are representative of at least two independent experiments with three animals per time point. **(E)** Microarray evaluation of mRNA expression levels of selected genes. For sorts, material from 8 to 15 mice was pooled in each case before RNA was isolated and processed. Data source [and Gene Expression Omnibus (GEO) accession number] as described in the text and in Section “Materials and Methods.” RU represents relative expression strength. Shown are mean ± SD (*n* = 2 in PP and d25, and *n* = 3 in d12). Data in **(A,B)** are from B6 mice and in **(C–E)** from Foxp3 mice. One-way ANOVA followed by Tukey’s *post hoc* analysis was performed in **(E)**. N/A: not applicable, ns: not significant (*p* > 0.05), *p < 0.05, **p < 0.01, and ***p < 0.001.

**Figure 2:**
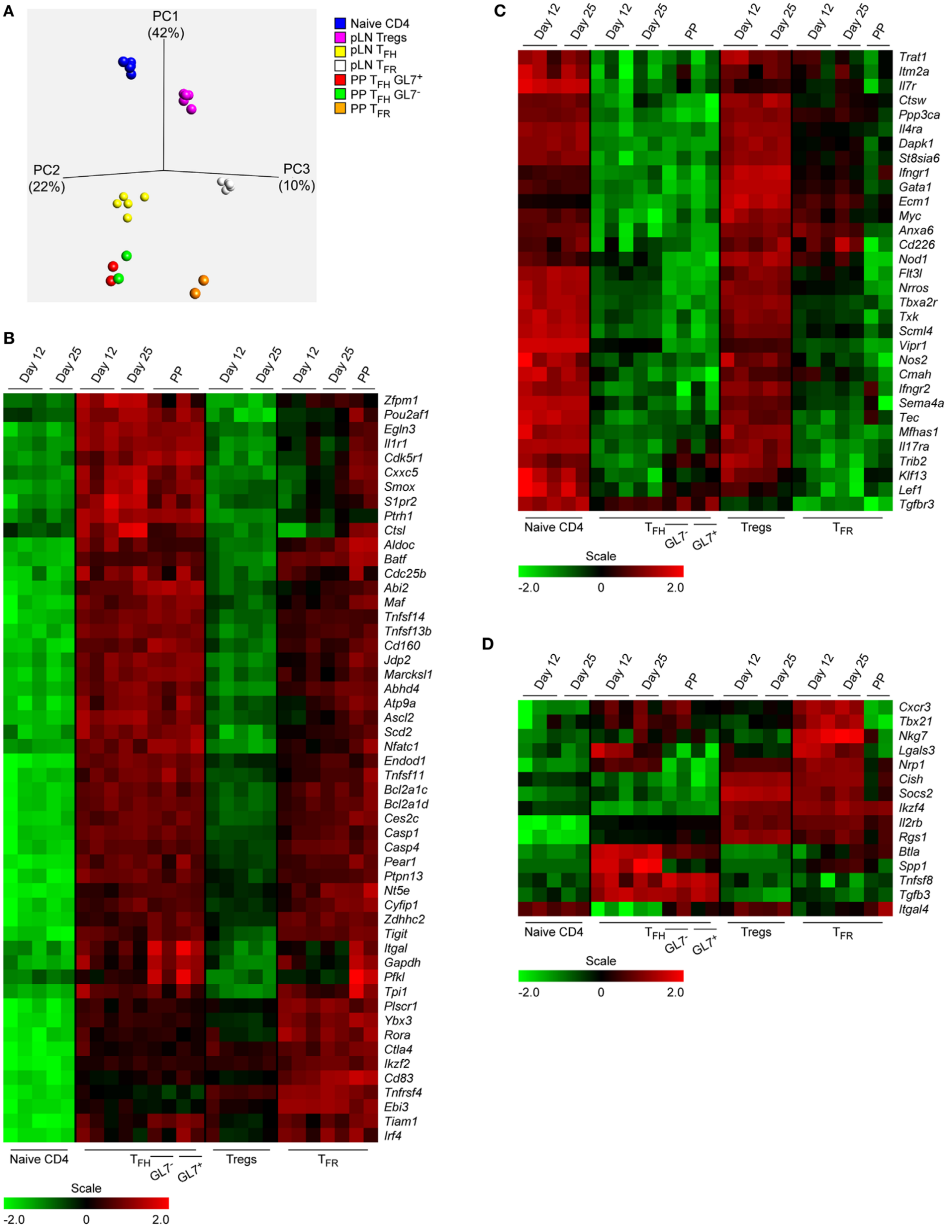
Expression patterns of CD4 T cell subsets from Peyer’s Patches (PP) and immunized peripheral lymph node (pLN). **(A)** A PCA analysis was done based on the expression of 8,359 most differentially transcribed genes with *p* < 0.001 and a *q*-value cutoff 0.005 with the *q*-value describing the false discovery rate. Definition of the cell subtypes as described in the text. **(B–D)** Heat maps illustrating regulation of mRNA expression of selected genes listed to the right. Same data source as in Figure 1E.

**Figure 3:**
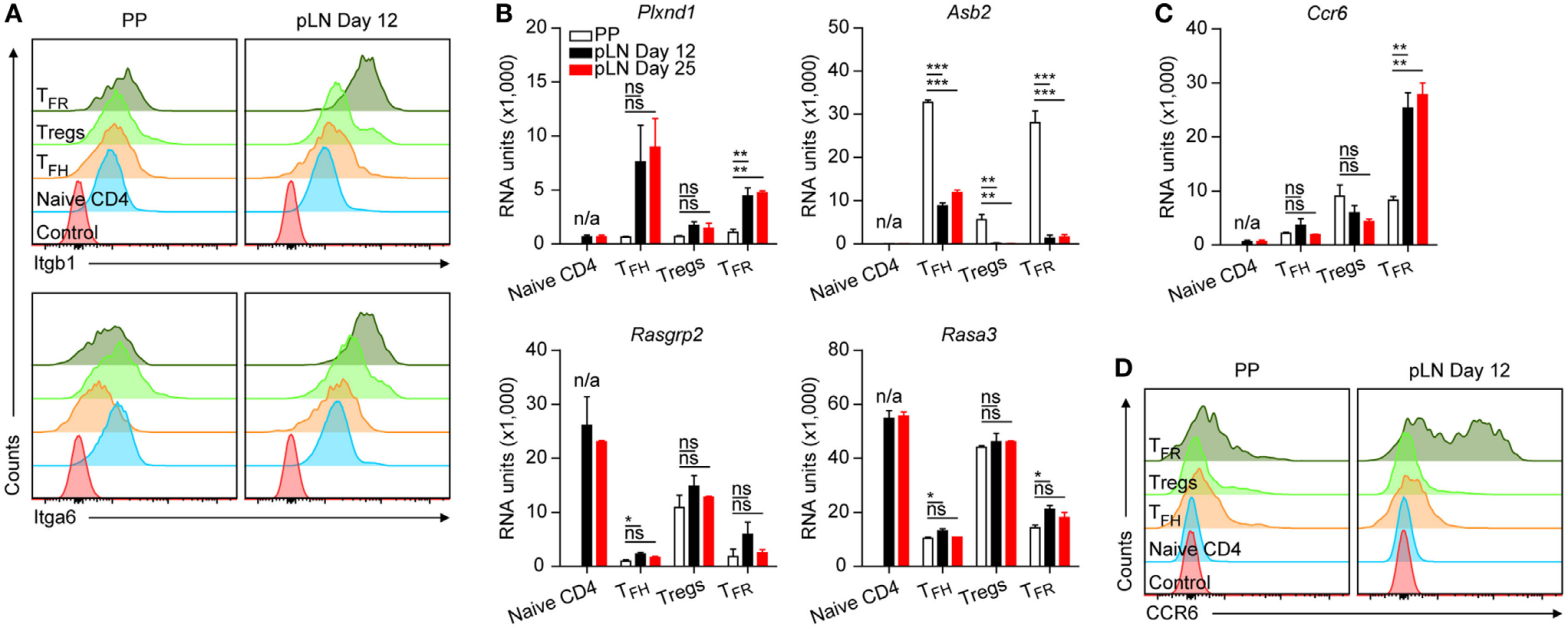
Protein and mRNA levels of selected genes in CD4 T cell subsets. **(A,D)** Surface expression of integrin α6 and β1 as well as CCR6 on the indicated cell types from Peyer’s Patches (PP) and peripheral lymph nodes (pLNs) at d12 p.i. with KLH + Alum. Data are representative of at least two independent experiments with *n* ≥ 2 animals per experiment. **(B,C)** Microarray data for mRNA expression levels of the depicted genes. Same data source as in Figure 1E. Shown are mean ± SD. One-way ANOVA followed by Tukey’s *post hoc* analysis was performed in **(B,C)** N/A: not applicable, ns: not significant (*p* > 0.05), *p < 0.05, **p < 0.01, and ***p < 0.001.

**Figure 4:**
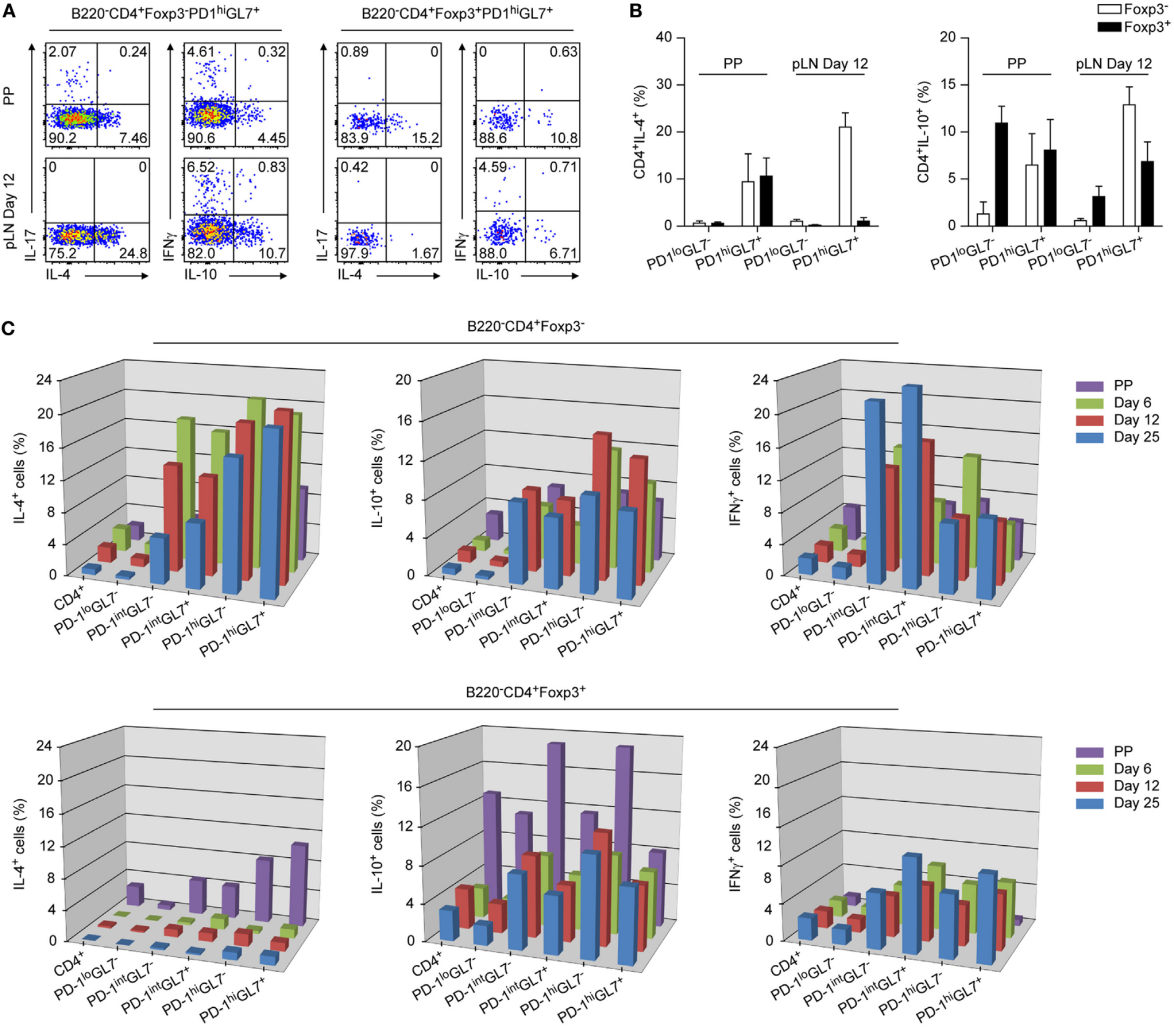
Cytokine profiles of TFH and TFR cells from Peyer’s Patches (PP) and peripheral lymph node (pLN). Total cell preparations from PP and pLNs d6, d12, and d25 p.i. were *in vitro* stimulated with PMA/ionomycin in the presence of Brefeldin A, followed by intracellular detection of the indicated cytokines by flow cytometry. **(A)** Representative plots depicting expression of IL4, IL10, IL17, and IFNγ by TFH and TFR cells of PP or pLN 12 days p.i. as indicated are shown. **(B)** Summary of **(A)** (shown are mean ± SD). **(C)** 3D representation of time kinetics of cytokine production by pLN TFH and TFR cells from d6, d12, and d25 p.i. with KLH + Alum. Data from PP cells are also shown. Frequencies of positive cells are measured *via* intracellular detection of cytokines by flow cytometry as in **(A)**. Data were combined from two independent experiments with two mice per experiment for each time point. Shown are mean values.

**Figure 5:**
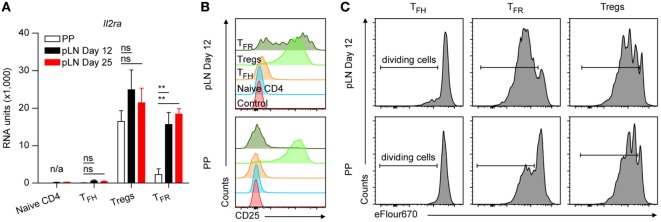
IL2 responsiveness of TFR cells. **(A)** Microarray data for mRNA expression levels of *Il2ra*. Same data source as in Figure 1E. Shown are mean ± SD. **(B)** Expression levels of CD25 on the indicated cell types from Peyer’s Patches (PP) and peripheral lymph nodes (pLNs) d12 p.i. with KLH + Alum. Shown are representative flow cytometry histograms of two independent experiments with *n* = 6. **(C)** Cell proliferation assay of sorted cell types as indicated, labeled with cell proliferation dye and *in vitro* cultured on anti-CD3 coated plates in presence of IL2. Shown are representative flow cytometry plots of two independent experiments with pooled material sorted from eight mice per experiment. One-way ANOVA followed by Tukey’s *post hoc* analysis was performed in **(A)**. N/A: not applicable, ns: not significant (*p* > 0.05) and **p < 0.01.

**Figure 6:**
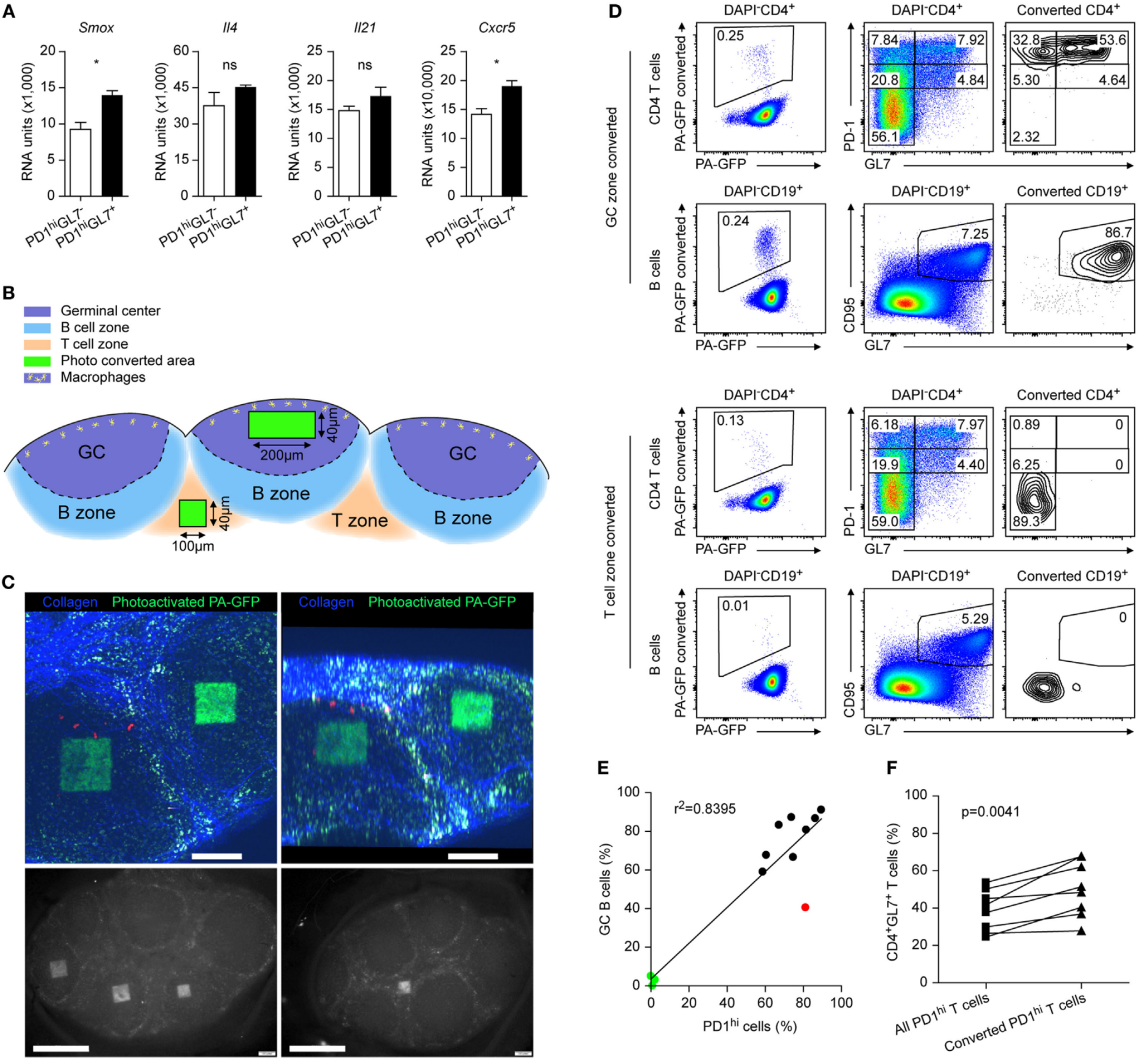
Reviewing GL7 as marker for follicular T cells. **(A)** Microarray data from Peyer’s Patches (PP) for mRNA expression levels of the depicted genes in PD1^hi^GL^−^ (open bars) versus PD1^hi^GL7^+^ (black bars) TFH cells. Same data source as in Figure 1E. Shown are mean ± SD. **(B)** Sketch of the two-photon converted regions. **(C)** Pictures of the converted regions inside the PP germinal center (GC) by two-photon microscopy (upper panels) and by epifluorescence microscopy (GC conversion lower left panel and interfollicular region lower right panel). Scale bar = 200 (upper panels) and 500 µm (lower panels). **(D)** Flow cytometry analysis of the illuminated PP with the converted cells boxed in the left panels. Gating was done as indicated. **(E)** Correlation between frequencies of GC B cells among all converted B cells and the frequencies of PD1^hi^ cells among all converted CD4^+^ cells. Each dot represents a separate PP investigated. Black dots: conversion focused on GC, green dots: conversion focused on interfollicular regions, red dot: excluded from the analysis in **(F)** due to the low frequency of GC B cells. **(F)** Direct comparisons of frequencies of GL7^+^ cells among all PD1^hi^ cells to those located only in converted GC of the same PP. **(C–F)** Data are from three independent experiments (*n* = 3). Unpaired two-tailed *t*-test was performed in **(A)** and paired two-tailed *t*-test was done in **(F)**. ns: not significant (*p* > 0.05) and *p < 0.05.

**Figure 7:**
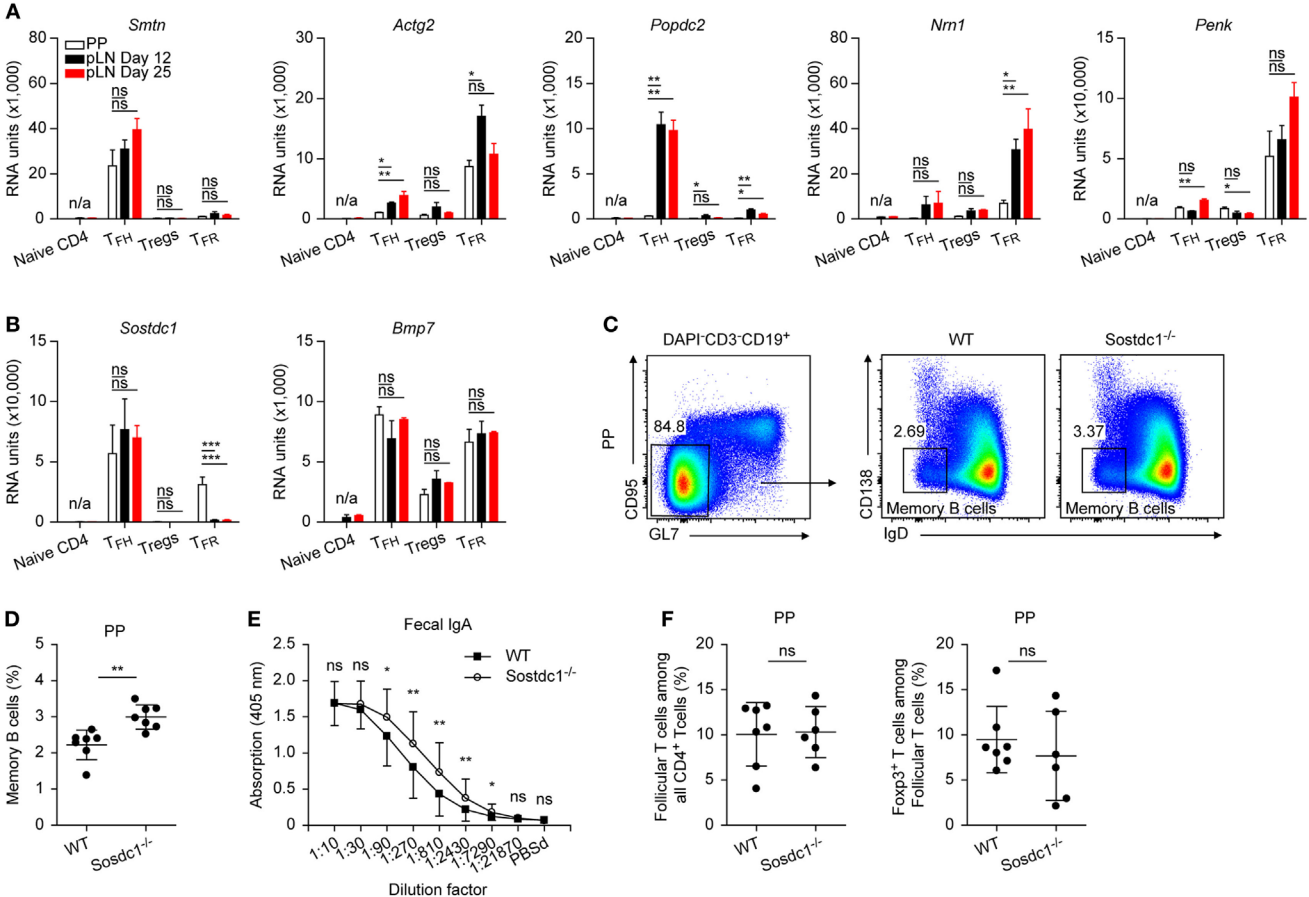
Further genes of interest. **(A,B)** Microarray data for mRNA expression levels of the depicted genes. Same data source as in Figure 1E. **(C)** Gating strategy illustrating definition of memory B cells in Peyer’s Patches (PP). **(D)** Data summary showing frequencies of memory B cells in PP of Sostdc1^+/+^ and Sostdc1^−/−^ mice. Each dot represents one mouse. Shown are mean ± SD. **(E)** Determination of fecal IgA by ELISA, samples were tested in duplicates. Mean ± SD are given for each dilution step. **(F)** Left panel: Frequencies of PD1^hi^ follicular T cells among all CD4^+^ cells in PP of Sostdc1^+/+^ and Sostdc1^−/−^ mice according to the gating criteria shown in Figure 1A. Right panel: Frequencies of TFR cells among follicular cells of PP. Foxp3 was detected by intracellular stain as described in Section “Materials and Methods.” Shown are mean ± SD. One-way ANOVA followed by Tukey’s *post hoc* analysis was performed in **(A,B)** Data from three independent experiments (*n* = 7) were pooled and two-tailed *t*-tests were done in **(D–F)**. N/A: not applicable, ns: not significant (*p* > 0.05), *p < 0.05, **p < 0.01, and ***p < 0.001.

The heat maps and the PCA analysis shown in Figure 2 were generated by Qlucore Omics Explorer software tool from the normalized gPS values for selected genes. The *n* values equal 4–7 animals per group in Figures 1A–D, 3A,D, 4, 5B, and 7C–F, and three animals in Figures 6C–F. In Figures 6E,F, each dot represents single PP. For the transcriptome analysis (Figures 1E, 2, 3B,C, 5A, 6A, and 7A,B; Figures S1C, S2, and S4 in Supplementary Material), each cell fraction (where *n* = cell fraction) is sorted from pooled organs harvest from 7 to 8 mice (*n* = 2 in PP and day 25, and *n* = 3 in day 12). All animals in this study were analyzed at the age of 8–14 weeks old. Mean values of each group are shown in all figures and error bars represent ± SD. All statistical analyses were performed with GraphPad Prism 4. One-way ANOVA followed by Tukey’s *post hoc* analyses were performed in Figures 1E, 3B,C, 5A, and 7A,B; Figures S1C, S2 and S4 in Supplementary Material. Unpaired two-tailed *t*-test was done in Figures 6A and 7D–F and paired two-tailed *t*-test was done in Figure 6F as indicated in the legends of the figures.

## Results

### Flow Cytometry of Follicular CD4 T Cells in PP and pLN

Gating of CD4 T cells of PP using standard markers PD1 and ICOS or CXCR5 discriminated three distinct populations: PD1^lo^, PD1^int^, and PD1^hi^ (Figure 1A), a pattern highly similar to that found in human tonsils (25). Whereas populations in gates 2 and 3 appeared uniform under these conditions, a stain including the carbohydrate epitope GL7 allowed a further dissection into GL7^−^ (2a, 3a) and GL7^+^ (2b, 3b) subpopulations (Figure 1A). The GL7 epitope was suggested to be present only on TFH cells inside the GC (26) but it is expressed by CD4 T cells already early following immunization at times when GC do not yet exist (27). Therefore, PD1^int^GL7^+^ cells most likely represent preTFH cells whereas the PD1^int^GL7^−^ pool consists of additional cells such as Treg and effector helper cells. In support of this, only few PD1^int^ cells were detectable in the GL7^+^ gate in steady state pLN (Figure 1B, d0) but came into existence upon immunization (≥d6). In general, the very same gating strategy as applied for PP can be used to identify TFH or preTFH cells in immunized pLN of B6 mice (Figure 1B). On d6 post immunization (p.i.), a prominent PD1^hi^GL7^−^ population was formed but diffuse PD1^int/hi^GL7^+^ clusters were also present. At d12, a distinct PD1^hi^GL7^+^ population emerged whereas less PD1^hi^GL7^−^ cells existed. This distribution remained unchanged at d25 but less PD1^int^GL7^+^ cells were present.

A considerable proportion of CD4 T cells consist of Treg cells. Therefore, the strain C57BL/6-Foxp3^EGFP^ (Foxp3 mouse) (28) was used allowing a separate analysis of Foxp3 expressing cells. The PD1/GL7-distribution pattern of PP CD4^+^Foxp3^+^ cells resembled remarkably that of the CD4^+^Foxp3^−^ cells (Figure 1C). Again, PD1^hi^ cells were virtually undetectable in non-immunized pLN (Figure 1D) and also CD4^+^GL7^+^ cells were only rarely observed. However, a small but noticeable fraction of PD1^int^ Treg cells was found representing activated cells (29).

### Transcriptome Analysis of CD4 Follicular T Cells

To elucidate potential differences between follicular T cells in pLN versus PP, transcriptome analyses were done by sorting cells from PP or pLN at d12 and d25 p.i. of Foxp3 mice. A gating strategy applied as shown in Figures 1C,D was used to obtain GFP^−^PD1^lo^GL7^−^ (naïve T), GFP^+^PD1^lo^GL7^−^ (Treg), GFP^−^PD1^hi^GL7^+^ (TFH), and GFP^+^PD1^hi^GL7^+/−^ (TFR) cells, respectively, from immunized pLN. Cells of PP origin were sorted accordingly but instead of naïve T cells, GFP^−^PD1^hi^GL7^−^ cells were isolated. RNA from sorted cells was processed, hybridized on Agilent chips (see Materials and Methods) and the results deposited in the GEO database (accession number: GSE106593). The purity of the sorts was usually ≥95% (Figures S1A,B in Supplementary Material) and all PD1^hi^ cells obtained by the applied gating strategy were also CXCR5^+^ (data not shown, see also Figure 1A). Purity is further illustrated by genes like *Lrrc32* that is expressed specifically by Treg cells (30) (Figure S1C in Supplementary Material). Inversely, expression of *Pde3b*, coding a cyclic nucleotide phosphodiesterase, is low in Foxp3^+^ cells. Its downregulation is controlled by FoxP3 and essential for Treg cell homeostasis (31). Typical signature genes regulated during differentiation of TFH and TFR cells display the expected pattern (Figure 1E) such as the coordinated switch in the *Ccr7*/*Cxcr5* balance. Downregulation of *Klf2* that would otherwise induce expression of *Sell* (CD62L), *S1pr1*, as well as *Selplg* (PSGL1) and repress that of *Bcl6* is a key event in the TFH pathway (11). A salient regulation was also observed for genes directly (e.g., *Ptpn11, Satb1, Stx11*) or indirectly (e.g., *Tbc1d4, Hif1a, Mafg*) affecting performance of T cells. IL17 plays a role in correct positioning of TFH cells in the light zone (32). This is mediated in part by IL17-driven upregulation of *Rgs16*, a regulator of G protein signaling rendering the cell unresponsive to CXCR4 triggered chemotaxis. In accordance with this, expression of *Sytl1* (SLP1) that promotes cell migration *via* the CXCR4/CXCL12 axis was downregulated in TFH cells. However, *Sytl1* was not downregulated in pLN TFR cells and only partially in PP TFR cells. *Tsc22d3* (GILZ) regulates TH17 cell differentiation (33) and its downregulation might influence development of IL17 producing TFH cells. This may also apply to the role of IL1 that is involved in TH17 differentiation (34). A delicate balance seems to exist regarding sensitivity to IL1 signaling since both, the functional *Il1r1* and the decoy receptor *Il1r2* are upregulated in TFH and TFR cells yet to a diverging extent depending on time and location.

A PCA analysis of the transcriptomes that is based on over 8,000 most diversely transcribed genes illustrates that follicular T cells clearly differ from their progenitors (naïve T and Treg cells) but also indicates that neither TFH cells nor TFR cells of pLN and PP origin cluster together. However, TFH cells of both compartments appear more closely related to each other than the corresponding TFR cells (Figure 2A). The heat maps shown in Figures 2B–D depict genes exerting considerable regulation of their expression during differentiation into follicular T cells. Only exemplary genes were selected where published evidence supports a role in T cell functionality or TFH cell differentiation and/or function. The genes listed were grouped into those that were found to be upregulated (B) or downmodulated (C) in follicular cells. A third category of genes (D) did not obey a simple regulatory scheme.

### Delayed or Attenuated Regulation of Genes in Follicular T Cells

A coherent degree of regulation was observed for TFH and TFR cells of PP as well as pLN origin in many cases (Figure 1E, e.g., *S1pr1, Satb1, Ptpn13, Ccr7, Tsc22d3*). However, the expression profiles also revealed diverging tendencies for many genes. Although regulation of these genes in TFH cells was in general similar in extent in cells of PP and pLN origin (Figures 1E and 2B,C), the degree of regulation in TFR cells was heterogeneous depending on their origin. Except for rare cases (e.g., *Ptpn11* and *Cxcr5*), PP TFR cells resembled PP TFH or pLN TFH cells. Yet, a broad spectrum of genes displayed a significantly smaller degree of regulation in pLN TFR cells when compared to PP TFR cells and this discrepancy continued to exist in d25 cells (e.g., *Bcl6, Stx11, Gadd45b, Hif1a, Cxcr5, Rgs16, Il1r1*). In some instances, however, regulation of genes in d25 TFR cells reached the level met in PP TFR cells (e.g., *Klf2, Sell, Tbc1d4*). The heat maps in Figures 2B,C visualize further examples of complete versus incomplete or delayed regulation in TFR cells of pLN origin. Along with the global PCA analysis (Figure 2A), this suggests not only a retarded differentiation schedule generating matured pLN TFR cells (as compared to PP TFR and PP/pLN TFH cells) but also an attenuated regulation depending on the particular gene investigated. This may manifest itself in specific variations in functionality. For instance, it was proposed that a low level of CXCR5 expression by TFR cells localizes them more to the border of the GC (35).

### Genes Expressed Differently by CD4 T Cells From PP and pLN

We identified genes that are expressed in a compartment specific manner and affected not only TFH and TFR cells but also Treg cells (Figure S2 in Supplementary Material). *S100a4, Gcnt1* (C2GNT, a glucosaminyl transferase), and *Fut7* (FUC-TVII, fucosyl transferase 7) were expressed predominantly by pLN cells whereas PP cells expressed mainly *Dhrs3* and *Sun5*. Activity of C2GNT and FUC-TVII is required for formation of a functional P/E-selectin ligand in T cells (36). Despite downregulation of *Selplg* (PSGL1) in TFH/TFR cells (Figure 1E) a considerable basal level of expression persisted. Thus, the absence of FUC-TVII and C2GNT in PP cells would render any remaining PSGL1 driven binding activity to selectins non-functional. Interestingly, the vitamin A metabolite all-trans retinoic acid (RA) produced abundantly by intestinal cells counteracts *Fut7* expression but induces that of *Dhrs3* that is involved in RA metabolism. In addition, vitamin D impairs formation of selectin ligands (36) and expression of *Vdr* (vitamin D receptor) is upregulated in TFH cells and PP TFR cells. This suggests that local availability of compounds like vitamins influence the function of follicular but also Treg cells.

Peyer’s Patches T cells had uniformly low levels of *Itgb1* (CD29) whereas among pLN cells expression of this integrin chain was heterogeneous (Figure S2 in Supplementary Material). Flow cytometry confirmed that the vast majority of PP cells expressed moderately this integrin on the surface whereas in pLN TFH and predominantly TFR cells expressed higher levels (Figure 3A; Figure S3 in Supplementary Material illustrates gating defining the cell subtypes). Also with regard to *Itga6* (CD49f), PP TFH and TFR cells expressed much less CD49f complexed integrin compared to their pLN counterparts (Figure 3A; Figure S2 in Supplementary Material). Integrin α6 can pair with either β1 or β4. The latter is hardly expressed suggesting that in pLN but not PP TFH cells and more prominently TFR cells express integrin α_6β1_. In parallel, only pLN follicular T cells upregulated *Plxnd1* (Plexin D1), a surface bound receptor that controls clustering of β1-integrins thereby manipulating their avidity (37) (Figure 3B). *Vice versa*, a conspicuous upregulation of *Asb2* was evident in PP cells, particularly in TFR cells. ASB-2 expression is sensitive to RA and enhances integrin-dependent adhesion by promoting degradation of filamin (38). Additional genes modulating integrin binding activity were also regulated substantially during differentiation of follicular T cells such as *Rasgpr2* (CalDAG-GEF) and *Rasa3*. These observations illustrate that integrin mediated functions drastically change during differentiation into TFH/TFR cells but also suggest that cells of PP and pLN origin differ from each other in these aspects. Moreover, a divergent upregulation of *Cxcr3* (Figure 2D) and *Ccr6* (Figure 3C) was detected in TFR cells of different origin. In periphery, the majority of TFR cells expressed high levels of CCR6 whereas PP TFR cells were CCR6^lo/−^ (Figure 3D).

### IL4 and IL21 Are Produced by PP TFR Cells

The cytokines IL4 (10) and IL21 (2, 12) are central players in the GC reaction and it was shown that they are expressed abundantly by TFH cells but not by TFR cells in periphery (18). In stark contrast, we found that PP TFR cells expressed substantial amounts of *Il4* and to a lesser extent also *Il21* (Figure S4A in Supplementary Material). This was confirmed by intracellular detection of IL4 following PMA/ionomycin stimulation (Figures 4A,B). In these flow cytometry based cytokine analyses we discriminated between GL7^+^ and GL7^−^ follicular cells and also performed a kinetic analysis determining cytokine production of T cell subsets at d6, d12, and d25 p.i. in pLN (Figure 4C). In periphery, highest amounts of IL4 were produced by TFH cells already at d6 p.i. but also PD1^int^ cells produced IL4 predominantly early following immunization. As expected, peripheral FoxP3^+^ cells were virtually negative for IL4 at all time points analyzed (18). The regulatory circuits controlling IL4 and IL21 expression in TFH cells encompass the transcription factors *Batf* (BATF1) and *Maf* (C-MAF) and in both cases we observed increased levels of expression in PP TFR cells (6, 9, 39) (Figure S4B in Supplementary Material). It was reported that the CNS2 enhancer of the *Il4* gene is of critical importance for transcription in TFH cells (40, 41) and it was demonstrated recently that BATF1 and C-MAF bind the CNS2 enhancer driving *Il4* transcription (39). In addition, expression of *Bach2* (BTBD25), a negative regular of BATF1 activity, is uniformly downregulated in follicular T cells (42). *Il4* expression is possibly supported in PP by abundant upregulation of *Cebpa* (43) whereas that of *Il21* may be fostered by IL27 (44) (Figure S4B in Supplementary Material). Elevated levels of *Lag3*, an IL27 responsive gene, may indicate increased availability of this cytokine in PP and also expression of *Il27ra* is higher in PP cells as compared to pLN cells supporting a role of IL27 in IL21 expression by PP TFR cells (Figure S4B in Supplementary Material).

IL10 is expressed not only by Treg cells but also by TFH and TFR cells (15, 18). Expectedly, PP Treg cells expressed more IL10 mRNA and protein than did pLN Treg cells (Figure 4; Figure S4A in Supplementary Material). IL10 expression culminated in peripheral TFH cells at day12 p.i. and declines thereafter (Figure 4C) whereas it is slightly increased in pLN TFR cells. Among follicular T cells, a low percentage of PP TFH cells produced IL17 (Figure 4A). This would be in line with a report describing that in PP but not periphery TH17 cells can develop into TFH cells (45).

A substantial expression of IFNγ was detected in PD1^int^ cells while IFNγ expression remained modest in PP/pLN TFH cells although levels increased over time in pLN (Figure 4C). Interestingly, in pLN but not in PP, a small fraction of TFR cells produced IFNγ (Figures 4A,C; Figure S4A in Supplementary Material). This parallels expression of *Tbx21* (TBET) in these cells (Figure 2D). Evaluating IL4 versus IFNγ expression, we noted an increasing preference toward IFNγ production over time in pLN and thus a TH1 phenotype among PD1^int^ cells as expected for a B6 background. But regarding PD1^hi^ TFH cells, a significantly higher proportion of IL4^+^ cells existed reflecting the superior importance of this cytokine in the TFH driven GC reaction.

### TFR Cells of pLN but Not of PP Origin Are IL2 Responsive

IL2 is a potent inhibitor of TFH cell differentiation (46) wherefore its signaling must be silenced. Consequently, as in naïve CD4 T cells, expression of the high affinity IL2 receptor *Il2ra* (CD25) is absent or very low in TFH cells. However, CD25 is massively expressed by natural Treg cells from which TFR cells are derived. CD25 expression was found on TFR cells yet at decreased (35) or heterogeneous levels (18). Recently, peripheral CD25^+^ and CD25^−^ TFR cells were analyzed separately suggesting that CD25^−^ TFR cells represent matured cells (47). We also observed robust downregulation of *Il2ra* by TFR cells of PP but not of pLN origin even not at day 25 p.i. (Figure 5A). Flow cytometry confirmed that a substantial proportion of pLN TFR cells kept high levels of IL2RA on their surface but only a minority of PP TFR cells were CD25^+^ (Figure 5B; Figure S3 in Supplementary Material). In contrast to the peripheral CD25^−^ TFR cells investigated by Wing et al. (47), PP TFR cells remained largely unresponsive to IL2 under stimulatory conditions but pLN TFR cells propagated to an extent as Treg cells (Figure 5C). The *Il2ra* expression pattern in the T cell subsets of interest here is similar to those observed for *Cish* and *Socs2* (Figure 2D) two important mediators of IL2 signaling whose expression is induced by IL2 and that were already observed earlier to be regulated in follicular T cells (47, 48). This suggests that the IL2 signaling pathway is operative *in vivo* in pLN TFR cells but not in the vast majority of PP cells.

### GL7 Expression Does Not Distinguish GC Resident Follicular T Cells

GL7 is considered as a marker distinguishing GC TFH cells (26). In PP, two distinct PD1^hi^ populations, GL7^−^ and GL7^+^, respectively, exist in parallel at any time wherefore both were isolated and compared by transcriptome analysis. We failed to detect genes uniquely expressed in either population; only marginal differences exist between the expression of given genes of both populations that rarely reached statistical significance (Figure 6A; for further examples Figures 2 and 4). Therefore, we investigated the GL7 expression status of GC resident PD1^hi^ cells. To this end, a mouse strain expressing a photoactivatable fluorescent protein was used (PA-GFP mouse). Intact PP were excised from the small intestine and a two-photon laser beam programmed to irradiate a predefined cuboid inside a GC or in the interfollicular T area (Figures 6B,C). Following photoconversion of the cells inside the cuboids, cells of the organs were analyzed by flow cytometry. The vast majority of GC-born photoactivated CD4 T cells were PD1^hi^ but many of these were GL7^−^ (Figure 6D, compare all cells displayed in the middle panels to converted cells only in the right panels). The percentage of CD4^+^PD1^hi^ cells correlated with that of CD19^+^CD95^+^GL7^+^ GC B cells (Figures 6D,E) except for one case (red dot, neglected in Figure 6F) confirming that either a GC (black dots) or the interfollicular zone (green dots) was irradiated. The latter were virtually devoid of CD4^+^PD1^hi^ cells or GC B cells (Figure 6D). Compared to all CD4^+^PD1^hi^ cells inside a PP, these observations suggest an enrichment of PD1^hi^GL7^+^ cells inside the GC (Figure 6F) but the high proportion of GC PD1^hi^GL7^−^ cells indicates that GL7 is not an exclusive GC T cell marker.

### Candidate Genes

We identified a couple of genes that may be considered of primordial interest for further studies because they displayed a high degree of regulation that is paired in most cases with a thus far unrecognized or unique expression in cells of hematopoietic origin (Figure 7A). Among these are *Smtn* (smoothelin) as well as *Actg2* (actin γ2), cytoskeletal proteins found mainly in muscle cells and that are highly upregulated in either TFH or TFR cells. Similarly, *Popdc2* (POP2) is upregulated in TFH cells yet not in those of PP. *Nrn1* (neuritin) and *Penk* (proenkephalin) represent two genes that were upregulated strongly and preferentially in TFR cells. Neuritin promotes axonal outgrowth and recently it was shown that another axonal guidance molecule, ephrin B1 is important for TFH cell function (49).

Also *Sostdc1* (USAG1), a BMP antagonist (50), is profoundly upregulated in TFH cells and additionally in PP TFR cells (Figure 7B). In PP, absence of *Sostdc1* increased frequencies of memory B cells (Figures 7C,D). Moreover, the fecal IgA content was elevated in *Sostdc1*^−/−^ mice (Figure 7E) but follicular T cell frequencies remained unaltered (Figure 7F). Interestingly, *Bmp7* is also upregulated in follicular cells (Figure 7B) and *in vitro* BMP7 interfered with antibody production by human B cells (51). This documents an influence of USAG1on humoral immune responses yet further work will be needed to understand the underlying regulatory mechanisms.

## Discussion

In search for genes important for differentiation and/or function of TFH and TFR cells, we focused on those displaying differential expression patterns compared to naïve CD4 T or Treg cells in PP and immunized pLN. This approach inherently neglects all genes where expression remains unchanged during follicular T cell development but nevertheless is important for TFH or TFR biology. Moreover, we investigated the transcriptomes of follicular T cells at rather late time points following immunization (d12 and d25) and not at earlier days (approximately at d7) as done in many other studies. We had chosen later time points for two reasons: (i) although detectable already at d6, we observed a separate and easy to gate PD1^hi^GL7^+^ population only at later time points and (ii) available data from literature indicated that upon immunization, the mass of TFR cells comes into existence later than the TFH cells (47, 52). Furthermore, we considered it important to apply a restrictive gating to focus on cells in most advanced stages of the follicular T cell pathway and to avoid contamination with cell types that express PD1 at intermediate levels (e.g., preTFH cells, exhausted cells) or that exist in pLN already in steady state (activated Treg cells). This strategy was corroborated by the results following photo conversion of GC resident T cells, i.e., fully mature follicular cells, showing that the vast majority were PD1^hi^ cells according to the gate criteria applied here.

Apart from well-studied genes central to TFH/TFR biology, we identified a collection of genes displaying a regulation that would bring in line their known effects in T cells with those required for follicular T cell development and/or function. For example, upmodulation was observed for *Ptpn11* (SHP2, Figure 1E) that mediates the inhibitory activity of PD1 (53). Upregulation of PD1 correlated with downmodulation of *Satb1* coding a chromatin organizer that represses PD1 expression in T cells (54). A concomitant expression of PD1 and other inhibitory receptors (e.g., *Ctla4, Btla, Cd160, Tigit*; Figure 2) over extended periods of time is associated with T cell exhaustion that would lead to a loss of effector functions in the long run (55). Exhaustion is provoked by a persistent antigen driven stimulation as met by T cells in the GC. Remarkably, exhausted CD4 T cells exist that produce IL10 as well as IL21 and that express BCL6. In CD8 T cells, expression of *Stx11* (syntaxin 11) that was upregulated in TFH and TFR cells, counteracts exhaustion (56). A trait follicular T cells may share with TH2 cells is the specific expression of *Ptpn13* (FAP-1) that limits Fas/FasL-mediated apoptosis (57). *Gadd45b* (MyD118) expression is rapidly induced upon activation of CD4 T cells and its deficiency impairs cytokine production (58). *Tbc1d4* encodes a Rab GTPase-activating protein involved in regulation of glucose uptake. A concurrent upregulation of *Hif1a* was found. HIF1α is a key factor regulating metabolism by upregulating expression of genes engaged in glycolysis (59). This was surprising because BCL6 was shown to suppress the expression of genes of the glycolysis program in effector T cells (60) and glucose metabolism of TFH cells is considerably reduced compared to TH1 cells (61). By contrast, we observed a marked upregulation of BCL6 target genes such as *Tpi1* (triosephoshate isomerase), *Gapdh*, or *Pfkl* (phosphofructokinase) in follicular T cells compared to naïve CD4 and Treg cells (Figure 2B) suggesting that HIF1α may override BCL6 mediated repression in these particular cases. Evidence was provided that aerobic glycolysis is required for efficient cytokine production by T cells (62) and an ongoing substantial glucose metabolism driven by HIF1α is further indicated by the notion that *Mafg*, a small MAF protein facilitating nuclear localization of HIF1A (63) is upregulated in follicular cells.

The results presented here revealed that the magnitude of gene regulation was very similar between TFH cells of PP and pLN although some variations and micro-milieu triggered divergences existed. Much more profound differences existed between the TFR cells of both compartments and one key feature of PP TFR cells is the almost complete absence of *Il2ra* expression and a concomitant unresponsiveness to IL2. It was found recently that signature genes (e.g., *Bcl6, Pdcd1*, and *Btla*) were less well up-modulated in CD25^+^ TFR cells suggesting that they represent an intermediate stage whereas CD25^−^ cells are matured GC resident TFR cells (47). Accordingly, we observed that regulation of some genes (e.g., *Klf2, Tbc1d4*) tended to completion at d25 but others did not follow such a scheme (e.g., *Cxcr5, Hif1a*) and also the signal for *Il2ra* remained high among d25 pLN TFR cells arguing against a progress in differentiation toward CD25^lo^ TFR cells. However, in pLN new Treg cells may be recruited continuously into the TFR pathway generating immature CD25^+^ cells, a process that would then not occur efficiently in PP in steady state with its well established chronic GC. At any rate, the existence of a high rate of CD25^hi^ cells discriminated the pool of TFR cells of pLN from those of PP. In addition, only the latter were found to express *Il4* and *Il21* substantially and along with other noticeable differences (e.g., *Vdr, Fut7, Ccr6, Nrn1*) this gives rise to profound functional disparities. Thus, most activated B cells in PP would perform a switch to IgA that is propelled mainly by TGFβ but also other factors such as IL10, RA, and IL21 (64). Efficient IgA class switch takes place outside the GC in the SED that is rich in TGFβ-producing DC (65). To accomplish migration to the SED and interaction with DC, preGC B cells must express CCR6. Interestingly, we observed that pLN but not PP TFR cells prominently expressed this chemokine receptor. Therefore, in PP the IgA class switching cells might escape a fine-meshed control by TFR cells as met in the GC.

Notably, a temporal asymmetry exists regarding GC performance in periphery: at early time points, a broader spectrum of GC B cells with regard to antigen affinity as well as more memory B cells are generated whereas later on preferentially high affinity plasma cells are formed (66). Considering a delay in numbers and maturity, this would fit to a role of TFR cells suppressing GC B cell responses where only high affinity B cells would be able to overrule the increasing inhibition at later stages of the GC reaction (67). If this hypothesis is correct, it would be difficult to reconcile the role of the rather mature PP TFR cells with the commonly accepted scenario that the commensal microbiota-driven (chronic) GC responses in the PP would allow continuous generation of low-affinity GC B cells (64). However, we found that PP but not pLN TFR cells are capable to produce IL4 and IL21 although this contradicts their classical role, i.e., suppressing production of these cytokines by TFH cells (20). Of note, TFH-born IL4 efficiently drives plasma cell differentiation (68) and IL21endowes GC B cells to overcome an imprinted inhibition of propagation by TFR cells (69). We, therefore, speculate that by using these unique features PP TFR cells permit to some extent the proliferation of low-affinity GC B cells directly contacting them. For this purpose, also moderate levels of IL21production could be effective since the cytokine would be available at higher local concentrations at the cell surface. Possibly, this also fuels autocrine mediated signaling. IL21 augments expression of *Bcl6* that in turn impairs that of CD25 (70), a mechanism that may reinforce the more matured phenotype of PP TFR cells. Moreover, our observation of propagation incompetent CD25^−^IL4^+^IL21^+^ TFR cells could help explain why TFR cells of PP but not those of periphery can readily convert into TFH cells because they anticipate these TFH key features (24).

Taken together, our results unraveled may new aspects in TFH and TFR cell biology. This applies to genes of general relevance for follicular T cell function such as *Hif1a, Il4, Il21*, or the GL7 epitope but also genes supporting local peculiarities in function (e.g., *Ccr6, Asb2, Il2ra*, integrins and modulators of integrin function, *Fut7*). Interestingly, we discovered also genes that are more actively expressed in either of the TFH and TFR follicular subtypes, respectively, and also a wealth of interesting candidate genes. However, the importance of these genes for follicular T cell biology awaits experimental validation although we were able to document an influence of *Sostdc1* on efficiency of memory B cell generation in PP.

## Ethics Statement

All experiments including animals were approved by the Lower Saxony State Office for consumer protection and food safety (LAVES), and were conducted according to MHH guidelines.

## Author Contributions

HG, RF, and GB designed experiments; HG, IR, SH, and GP performed experiments; HG and GB analyzed experiments and wrote the paper; BM and GL provided knockout mice.

## Conflict of Interest Statement

The authors declare that the research was conducted in the absence of any commercial or financial relationships that could be construed as a potential conflict of interest.
