# Supplementary material for: Shared and Unique Features Distinguishing Follicular T Helper and Regulatory Cells of Peripheral Lymph Node and Peyer’s Patches
Source: Front Immunol. 2018 Apr 9;9:714. doi: 10.3389/fimmu.2018.00714 (PMC5900012; doi:10.3389/fimmu.2018.00714)
Supplement: Supplementary file 1 [file Data_Sheet_1.PDF]

Supplementary Material:

Shared and Unique Features Distinguishing Follicular T Helper and Regulatory Cells of Peripheral Lymph Node and Peyer's Patches

Hristo Georgiev, Inga Ravens, Georgia Papadogianni, Stephan Halle, Bernard Malissen, Gabriela G. Loots, Reinhold Förster, and Günter Bernhardt*

*Correspondence:
Günter Bernhardt
Bernhardt.guenter@mh-hannover.de

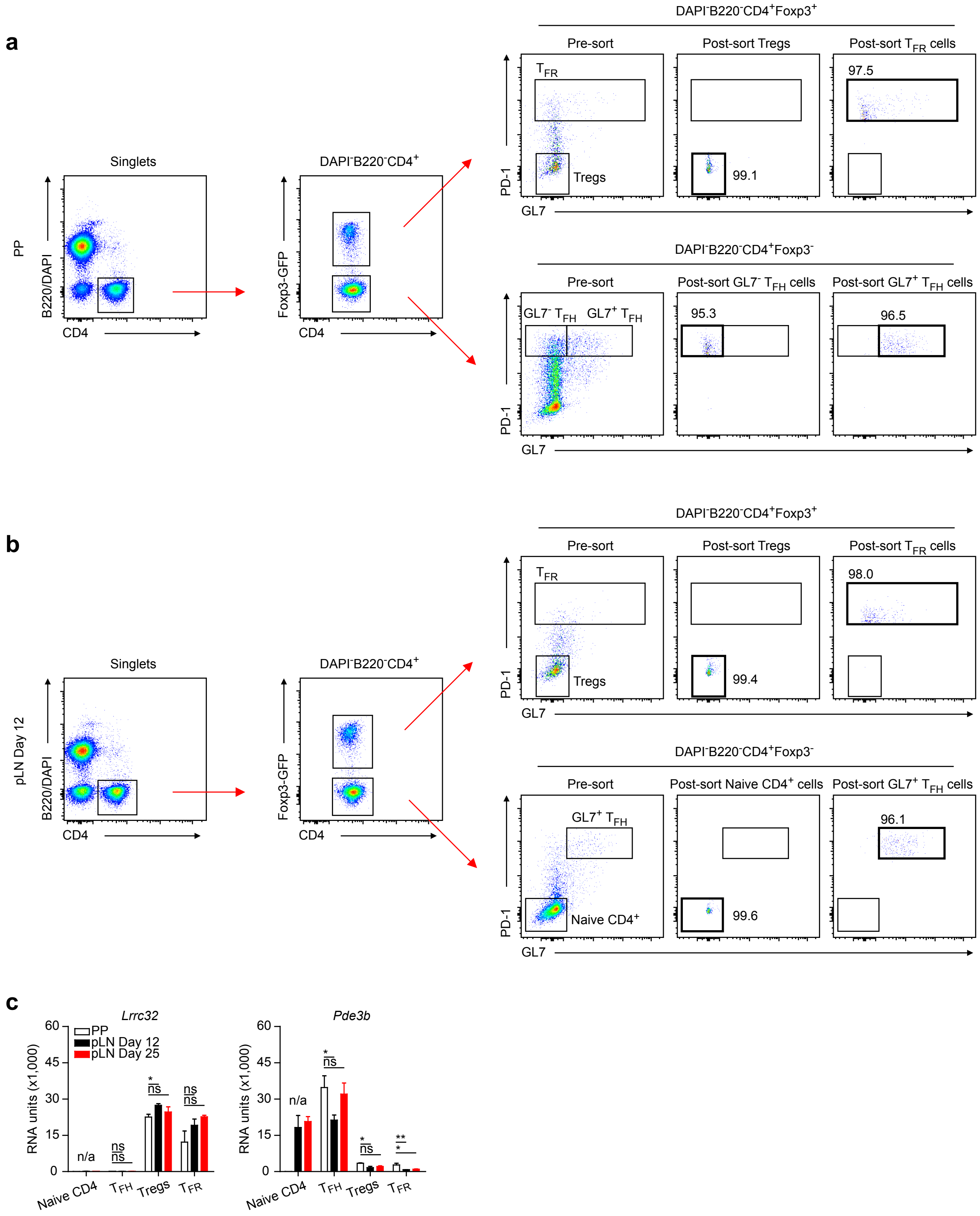

Supplementary Figure 1

Gating strategy for sorting T follicular CD4 cells from PP and pLNs.
(**A,B**) Sorting strategy of total cell preparations and the following purity reanalysis of the sorted fractions from PP (**A**) and pLN 12 days p.i. (**B**) with KLH+Alum. (**C**) Microarray data for mRNA expression levels of *Lrrc32* and *Pde3b*. Shown are means (±SD). Same data source as in **Fig. 1E**. One-way ANOVA followed by Tukey's post hoc analysis was performed in **C**. N/A: not applicable, ns: not significant ($p > 0.05$), *$p < 0.05$ and **$p < 0.01$.

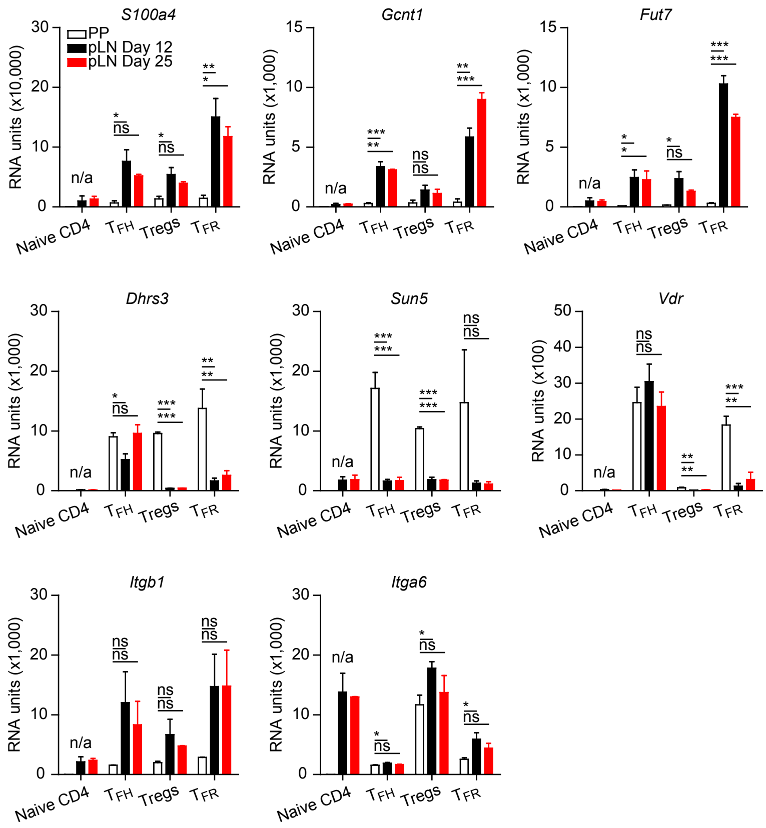

Supplementary Figure 2

Genes differently regulated between follicular T cells of PP and pLN.
Microarray data for mRNA expression levels of selected genes found to be differently expressed by the investigated CD4 T cell fractions from PP and pLN. Shown are means (±SD). Same data source as in **Fig. 1E**. One-way ANOVA followed by Tukey's post hoc analysis was performed in B. N/A: not applicable, ns: not significant ($p > 0.05$), *$p < 0.05$, **$p < 0.01$, and ***$p < 0.001$.

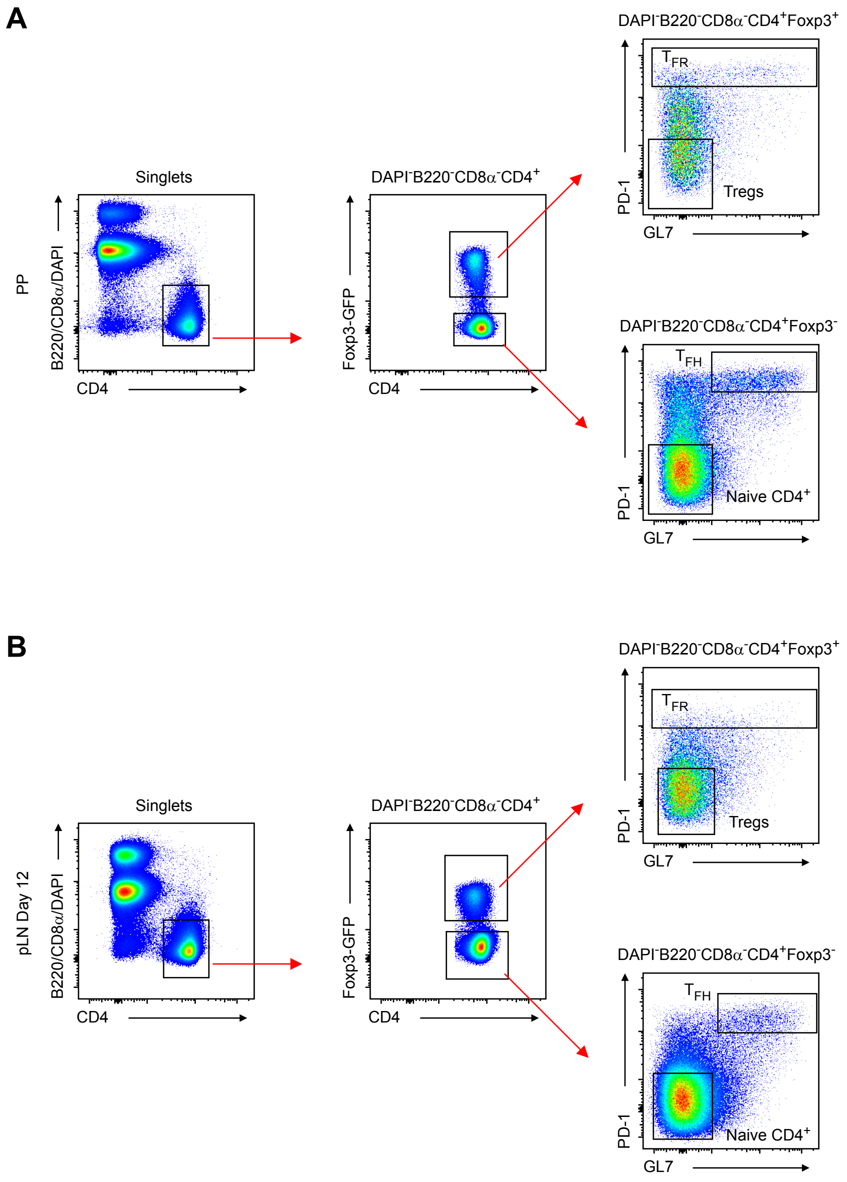

Supplementary Figure 3

Gating strategy for flow cytometry analysis of T follicular CD4 cells.
(**A,B**) Gating strategy applied for CD4 T cell subsets given in **Fig. 3A,D** and **5B**,**C**. Shown are representative FACS plots from PP in **A** and from pLN 12 days post immunization with KLH+Alum in **B**.

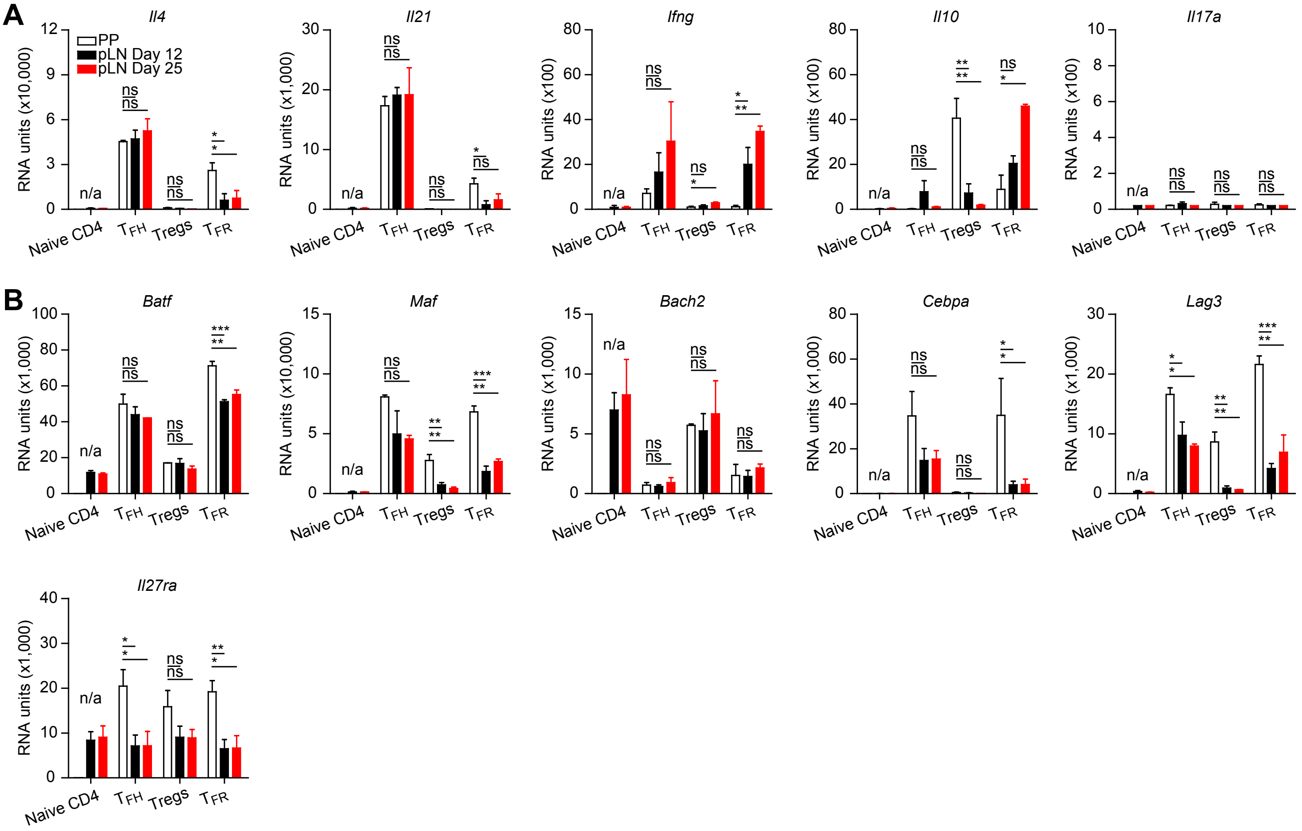

Supplementary Figure 4

Expression of cytokines and cytokine relevant genes.
Microarray data for mRNA expression levels of selected cytokines in **A**, and genes potentially involved in their regulation in **B**. Shown are means (±SD). Same data source as in **Fig. 1E**. One-way ANOVA followed by Tukey's post hoc analysis was performed in **B**. N/A: not applicable, ns: not significant ($p > 0.05$), *$p < 0.05$, **$p < 0.01$, and ***$p < 0.001$.
